# Current status of opioid epidemic in the United Kingdom and strategies for treatment optimisation in chronic pain

**DOI:** 10.1007/s11096-020-01205-y

**Published:** 2020-11-30

**Authors:** Aziza Alenezi, Asma Yahyouche, Vibhu Paudyal

**Affiliations:** School of Pharmacy, University of Birmingham, Birmingham, B15 2TT UK

**Keywords:** Chronic non-malignant pain, Chronic opioid therapy, Opioid use disorder, United Kingdom

## Abstract

The increase in opioid prescriptions in the United States has been accompanied by an increase in misuse as well as overdose and toxicity related morbidity and mortality. However, the extent of the increased opioid use, including misuse in the United Kingdom, currently remains less debated. Recent studies in the United Kingdom have shown a rise in opioid use and attributed deaths, particularly in areas with higher deprivation. There are also large variations amongst the devolved nations; Scotland has the highest drug-related deaths and year-on-year increase within Europe. Better clinical guidelines that can enable person-centred management of chronic pain, medicines optimisation, and early diagnosis and treatment of opioid use disorder are crucial to addressing opioid-related morbidity and mortality in the United Kingdom.

## Impacts on practice

- Triangulation of currently available data from multiple sources confirms that opioid crisis is deepening in the United Kingdom which needs urgent policy and practice focus on patient pathways to opioid misuse.
- Clinical guidelines and innovative service models that can enable person-centred management of chronic pain, medicines optimisation, and early diagnosis and treatment of opioid use disorder are needed.
- There is a need for better linkage of data across sectors to enable epidemiological surveillance and monitoring of prescribed and illicit opioids use.

## Introduction

The term ‘opioid epidemic’ specifically refers to increased deaths and hospitalisations due to prescribed and illicit opioids and their analogues. The opioid epidemic in the United States (US), [1] has led to calls internationally regarding rational prescribing and use of prescribed opioids as well as the need to address the misuse of illicit opioids (for instance, heroin and opium) and diverted pharmaceutical opioids (such as buprenorphine, methadone, and morphine). The aim of this paper is to critically discuss current practice and research regarding the extent of prescribed opioid use, particularly in the context of chronic non-malignant pain (CNMP) management, and identify how rising opioid (both prescribed and illicit use) related morbidity and mortality can be addressed.

### Chronic opioid therapy (COT) in the management of CNMP

COT defined as the use of opioids on most days for more than three months, remains the mainstay of CNMP [2]. CNMP is a condition where patients persistently or recurrently experience pain for at least three months [3]. CNMP affects 20% of adults worldwide. In the United Kingdom (UK), studies suggest that up to half of the population (about 28 million adults) may be living with CNMP [4]. There is, however, a lack of evidence of improved patient outcomes in relation to the use of COT in CNMP. A recent randomised clinical trial has shown adverse effects and notably worse pain intensity in opioid users compared to non-opioid users, with no difference in pain interference [5]. Evidence also suggests that discontinuing COT in CNMP does not substantially reduce pain intensity [6]. COT is associated with various adverse effects including suicide attempts and polydrug use (opioids with alcohol or benzodiazepines) serious fractures, immunosuppression and increased risk for cardiovascular disease and early mortality. Adverse consequences of COT on the healthcare system and the wider society due to opioids dependence, misuse, abuse and addiction have also been reported [7]. The term Opioid Use Disorder (OUD) is often used to define the clinical implications of COT. OUD is compulsive behaviour characterised by consumption of more opioid or for longer than originally intended that leads to serious impairment or distress [8].

In addition to CNMP, prescribing of opioids for acute indications such as surgical pain in opioid-naïve patients can also lead to a transition to COT. Data show that patients prescribed opioids within seven days of surgery are 44% more likely to become COT users within a year than those not prescribed an opioid [9].

### Trends in prescribed opioid use in the UK

Many UK based studies show an upward trend in opioid prescribing and use in the National Health Services (NHS) parallel to that in the US. In 2017–2018, 12.8% of England’s population had an opioid prescription dispensed, with approximately 50% taking them for at least 1 year [10]. A 400% rise in prescription opioids has been reported in the past decade in the UK [11]. Dispensing of opioids by community pharmacies increased in England from 33.1 million to 40.5 million between 2008 and 2018 [10].

Currently, in England, over half of drug deaths are known to involve an opiate [12]. Direct links between prescribing practices (including any potentially inappropriate prescribing) and the opioid related harm and deaths need to be further investigated. However, the observed increase in prescription opioids corresponds to increased opioid -related death trends. Overall, prescribed opioid associated deaths in England and Wales had a four-fold increase between 1993 and 2017 [12].

There is also an increasing trend in deaths associated with more potent opioids such as fentanyl products. For example, in England fentanyl-related deaths increased from eight in 2008 to 135 in 2017 [13]. Guidelines recommend that such strong opioids be prescribed for unremitting cases, specifically for short-term use only, stepping patients down to weaker opioids as appropriate, or withdrawing if ineffective [14]. However, prescribed doses are known to be too high and prescribed for too long. The rise in tramadol-related mortality combined with a rise in tramadol’s prescription supply [15] illustrates the link between prescribing and opioid-related morbidity and mortality.

### Geographical variations and link with deprivation

There appear to be regional variations within the UK in the prevalence of opioid use and deaths. Drug use in Scotland is disproportionately higher than in other parts of the UK. Between 2017 and 2018, Scotland had 1187 -related deaths per million populations, three times that in England and Wales [16]. Regional variations in higher opioid-related deaths have been linked to higher deprivation, social issues, and the availability of street fentanyl. Inequality-associated social risk factors (childhood drug exposure, prison drug exposure, adverse childhood events, homelessness, unemployment, social marginalisation, mental illness, psychological trauma) are other linked risk factors [16, 17].

Links between deprivation and higher rates of both prescribed and illicit opioid use have been further demonstrated in the published literature. Opioid prescriptions are nearly two times higher in areas with greater deprivation in England [17]. Approximately 90% of the highest opioid prescribing areas are located in the north of England, demonstrating a strong link with deprivation [18]. The greater prevalence of chronic pain and lack of adequate addiction treatment services in socio-economically deprived areas may lead to suboptimal prescribing practices. In addition, populations in deprived areas are more likely to take these medicines chronically, usually alongside antidepressants, benzodiazepines, or gabapentinoids [8, 10].

### The need for better diagnosis and linked datasets

Better use and linkage of healthcare databases are needed in the UK to obtain a clearer picture of the extent of both prescribed and illicit opioid use. There is a need to establish linked datasets to identify the magnitude of inappropriate including overprescribing, opioid abuse, diversion, adverse events including mortality, and underutilisation of substitution treatments for pain including CNMP and to develop further evidence-based approaches to addressing the problem. For instance, prescribed opioids can be obtained from various sources (the independent health sector and the internet) but data from these sources are not integrated. In addition, there is a lack of data on the sales of over-the-counter (OTC) opioid-containing preparations. The lack of integrated healthcare data between primary (general practice/family physician clinics) and secondary (hospital) is also a barrier to treatment optimisation. For example, National Health Services primary care prescribers face challenges due to delayed or incomplete patient discharge medical records from hospitals. There is also a need to improve toxicological assessments to identify causative factors for overdose incidents, information-sharing on drug overdose and deaths and establishment of useful links between national datasets, including death registrations and national treatment monitoring systems.

### Role of healthcare professionals and the need for person-centred guidelines

Person-centred care enhances the patient’s involvement in their own care to meet their unique expectations in treatment, planning, and delivery elements. Non-consideration of patient needs can lead to suboptimal outcomes. For example, in the US, the misapplication or misinterpretation of the Centre for Disease Control guideline on opioid prescribing for chronic pain led to forced opioid tapering and abandonment for patients who were on COT. Many CNMP patients faced a significant challenge in obtaining proper care, reportedly resulting in profound physical, emotional, and societal costs. Such incidents reinforce the need for patient-centred guidelines. Best practices in CNMP management should include developing an effective pain treatment plan after accurate evaluation to establish a diagnosis, with measurable outcomes that focus on improving patients’ quality of life (QoL), functionality, and daily living activities [19]. Also establishing a therapeutic alliance between patient and clinician based on compassionate, empathetic care is necessary to counter the suffering of CNMP patients.

One way to reduce the increase in opioid use is by re-evaluating and leveraging the existing healthcare professionals’ (HCPs) roles [19]. Health professionals’ ability to balance CNMP treatment while minimising risks for OUD is vital to reduce opioid-associated morbidity and prevent overdose. Appropriate initiation, reduction, or discontinuation of COT is essential to minimise patients’ risk of psychological distress and opioid-related hospitalisation. Due to the paucity of essential data surrounding the best approach to support COT patients or to treat OUD, a single clinician is unlikely to have the skills to manage these patients’ needs. A multidisciplinary team (MDT) is needed, combining the skills of diverse healthcare professionals including general practitioners, nurses, psychiatrists, pharmacists and pain consultants, moulding various interventions including medications and non-pharmacological interventions. MDTs can successfully reduce prescribed opioids, therefore associated disorders and have been identified to have greater long term effectiveness than usual care in reducing pain and disability, and improving QoL and ability to return to work [19].

The National Institute of Clinical Excellence (NICE) in England is aiming to publish an updated guideline on the management of CNMP, which aims to include rational prescribing of opioids. However, the problem has not been addressed optimally as these guidelines do not clearly state care and support for practical daily decisions in management. Individualised patient-centred care in the diagnosis and treatment of OUD related to CNMP is fundamental to improve the outcomes of treatment in these populations. Safe opioid stewardship, with regular re-evaluation of patient risk, is essential, which involves identifying risk factors from patient history, family history, current biopsychosocial factors, screening and diagnostic tools, and integrated behavioural, complementary interventions.

Improving access to services is vital in addressing opioid misuse. Access is often difficult for people with dual diagnoses of mental health problems and substance misuse and vulnerable groups such as the homeless populations [20, 21]. Therefore, primary care providers, particularly community pharmacists, play a vital role in facilitating accessibility. Patients presenting with a significant level of psychological, physical and/or socio-professional problems should be referred promptly to the appropriate service. Patients who repeatedly struggle with opioid abuse often end up homeless/imprisoned, less capable or motivated to improve their health because of their other life priorities [22, 23]. Healthcare professionals, including community pharmacists, must be supported in managing patients’ clinical and social needs, using innovative service models such as social prescribing [23, 24].

Many patients may find perceived stigma (disgrace, humiliation) and discrimination in healthcare settings as barriers to seeking treatment or optimising existing treatment for CNMP and may end up abusing opioids. As many patients who misuse prescribed medications often do not join the ‘typical’ subculture of illicit users (i.e. social elements associated with illicit drug consumption such as street consumption, socialising publicly with other illicit users) or/and do not seek help from addiction treatment services, their problems often go unrecognised and untreated [25]. In addition, numerous opioid-dependent individuals seek advice from the internet, rather than their HCP, often accessing unreliable source of information.

## Conclusion

Suboptimal management of CNMP remains one of the key issues linked to the rising trend in opioid prescribing in the UK. Evidence from research literature and UK national statistics suggests that such rising trends correspond to increasing opioid related harms and deaths. Opioid-related deaths are more prevalent in deprived areas with the North of England and Scotland in particular facing more significant problems. Better linkage of data across care sectors will enable enhanced epidemiological surveillance and monitoring to target the rising opioid-related morbidity and mortality and identify the extent of the contribution made by prescribed and illicit opioids use. There is an urge to enable healthcare professionals to manage CNMP better as well as to prevent, detect, and address OUD earlier. These could be achieved through better education and awareness of OUD, development of person-centred guidelines and a MDT approach combining pharmacological and evidence based non-pharmacological interventions. A comprehensive approach to manage the changing needs of CNMP patients will help them to achieve functional recovery, autonomy, and improved quality of life, preventing negative outcomes from their treatment.

## Data statement

All data generated or analysed during this study are included in this published article.
